# Association of breastfeeding duration with overweight and obesity among women in Ghana

**DOI:** 10.3389/fgwh.2024.1251849

**Published:** 2024-09-16

**Authors:** Derek Anamaale Tuoyire, Anthony Mwinilanaa Tampah-Naah

**Affiliations:** ^1^ Department of Community Medicine, University of Cape Coast, Cape Coast, Ghana; ^2^ Department of Geography, SD Dombo University of Business and Integrated Development Studies, Wa, Ghana

**Keywords:** obesity, overweight, breastfeeding, women, children, Ghana

## Abstract

**Background:**

There is a general concurrence on the health benefits that breastfeeding confers to children, including offering maximal protection against obesity across their life course. However, the scientific evidence on similar benefits for women who breastfeed their children remains inconclusive. This study contributes to the discourse by examining the association of breastfeeding duration with overweight and obesity among women in Ghana.

**Methods:**

Data on 8,516 women of reproductive age were pooled from the last five (5) Ghana Demographic and Health Surveys, and analysed using descriptive proportions and logistic regression models.

**Results:**

The prevalence of overweight and obesity was about 8% lower for women who breastfed their children beyond 18 months (overweight = 13%, obesity = 5%) compared with women who did not breastfeed (overweight = 21%, obesity = 13%) their children at all. With reference to women who did not breastfeed their children, a significant lower odds of obesity was observed for those who breastfed their children for 13–18 months (OR = 0.46, 95% CI = 0.268, 0.864) and >18 months (OR = 0.41, 95% CI = 0.216, 0.764), after adjusting for possible confounding factors.

**Discussion:**

Women who breastfeed their children for a minimum of 12 months have lower risk of developing obesity. Promoting prolonged breastfeeding among mothers could be an effective pathway to preventing obesity among women in Ghana.

## Introduction

International organisations including the World Health Organisation (WHO) and the United Nations Fund for Children (UNICEF) have been on the frontline of advocating for breastfeeding for children, especially exclusive breastfeeding for the first six months of the infant and then continued complementary feeding up to two years (1, 2). To ensure a smooth transition to successful breastfeeding, it is recommended that breastfeeding is initiated within one hour of birth (3). This promotes emotional bonding between the mother and the infant, and has a positive impact on breastfeeding duration. It has been observed that breastfeeding durations are longer in low- and middle-income countries compared to high-income countries (2).

The potential of breastfeeding in protecting women against overweight and obesity has equally been recognized but much less explored. Overweight and obesity have become a public health concern in countries worldwide, with recent rapid increases in prevalence among women of reproductive ages in low- and middle-income countries (4). Globally, about 40 percent of women are categorized as overweight and approximately 15 percent are obese (5). Overweight and obesity have become much more common in sub-Saharan Africa (6). For example, the Ghana Demographic and Health Survey (GDHS) reported an increasing trend in overweight and obesity from 25 percent in 2003, and 40 percent in 2014 to half (50%) of females in their reproductive ages (15–49 years) in 2022 (7–9). It is predicted that one in five women will be obese in 2030 (10). These observed increases in body mass index (BMI) of women have been linked to urbanization and unhealthy dietary habits (11). Nonetheless, epidemiological studies espouse the importance of breastfeeding in mitigating overweight and obesity in women (12).

Breastfeeding duration has been shown to minimize the accumulation of excessive fat in lactating mothers (13). Epidemiological data indicate that although a woman's body accumulates more fat during pregnancy, the increased metabolism needed to sustain breastfeeding helps to reduce the amount of fat stored during pregnancy (14–16). Less reduction in excess weight during postpartum periods contributes to extra fat accumulation in subsequent pregnancies leading to obesity (17). Yet, mothers who maintain continuous and longer breastfeeding durations have better chances of burning much of the fat accumulated during the pregnancy and postpartum period. Findings from studies show that the energy required by breastfeeding mothers to effectively produce breast milk is about 2.8 megajoules (670 kcal) per day, and out of this, approximately 2.1 megajoules (500 kcal) is obtained from foods consumed and the rest is obtained from fat stored during pregnancy (18). This level of energy expelled by breastfeeding mothers, therefore, suggests the practice of breastfeeding has an effect in reducing pregnancy related body fat accumulation.

The discourse on the association between breastfeeding duration and weight of mothers remains inconclusive. Although some studies point to a positive association between these parameters, almost all the available evidence emanate from more developed countries (19). For instance, Cieśla et al. (20) established an association between breastfeeding and an increase in body mass index in premenopausal women. On a similar tangent, Bobrow et al. (21) found that women who had ever breastfed during their reproductive period had significantly lower odds of being obese or overweight compared to those who never breastfed. Other recent studies (19, 22–24) have similarly established positive associations between breastfeeding duration and obesity among women. Conversely, some studies have demonstrated that (exclusive) breastfeeding was not associated with postpartum maternal weight or body fat (25–28). To the best of our knowledge and searches, there is the absence of empirical studies in Ghana lending evidence to the discourse on breastfeeding effects on women's body mass index. This study, therefore, contributes to the ensuing discourse by examining the effect of breastfeeding duration on overweight and obesity among women in Ghana, based on pooled data from five (5) nationally representative surveys.

## Methods

### Study setting

The setting for the study is Ghana, which is located along the West African coast south of the Sahara with a total land area of about 238,537 square kilometers. Ghana currently has 16 administrative regions, with a population of about 32 million people (50.7% female vs. 49.3% male). With respect to health, Ghana has a functional public health delivery system with the Ghana Health Service (GHS) as the main government agency responsible for the delivery of health services to the citizenry from the community to the national level (9).

### Study design and data

This study is based on an analytical design involving an analysis of cross-sectional secondary data pooled from five (1993, 1998, 2003, 2008 and 2014) GDHSs (29). The premier 1988 GDHS was excluded from the analysis mainly because anthropometric data (height and weight) required for the current analysis were not collected from respondents in that survey. The similarity in methodology (sampling, data collection, coding and cleaning) across the various GDHSs allows for data pooling and/or comparability of the surveys. A cross-sectional design was employed in all the surveys and the datasets are publicly available from http://dhsprogram.com/data/available-datasets.cfm.

Each survey employed a dual-staged stratified sampling approach, beginning with the random selecting of clusters drawn from enumeration areas (EAs) in each region based on probability proportional to population size. In the second stage of sampling, households were randomly sampled systematically from which women aged 15–49 years who consented to participate in the surveys were interviewed and the anthropometric measurements obtained. The interviews and anthropometric measurements were conducted by well trained personnel, with the latter undertaken in accordance with the standard MEASURE DHS Biomarker Field Manual (30).

### Study population and sampling

Given that the current study's focus is on the association of breastfeeding with overweight and obesity, the target population was women within their reproductive age (15–49 years) who had given birth within the last five years preceding each of the surveys. A total population of 29, 408 women were interviewed in the five GHDSs (1993 = 4,562, 1998 = 4,843, 2003 = 5,691, 2008 = 4,916 and 2014 = 9,396) from which the subsample for the current study was obtained. For the purpose of the current study, women aged 15–49 years who did not have a child in the last five years preceding each of the surveys and those with no corresponding information on both duration of breastfeeding and anthropometric measurements (height and weight) were excluded. In addition, women who were pregnant or lactating during the period of the survey were exluded. Hence, the total analytical sample for this study consisted of 8,516 women pooled from the five GDHSs (1993 = 1,674, 1998 = 1,936, 2003 = 2,143, 2008 = 1,767, and 2014 = 995).

### Dependent variable

The dependent variable (BMI status) was derived from the height and weight measures obtained from women in the surveys by first computing the BMI score (weight in kilograms divided by height in meters-squared) of each woman. Next, the BMI scores were categorized into underweight (BMI < 18.5 kg/m^2^), normal Weight (BMI of 18.5–24.9 kg/m^2^) overweight (BMI of 25.0–29.9 kg/m^2^) and obese (BMI ≥ 30.0 kg/m^2^), which are in accordance with the WHO standard BMI cut-offs (31). In line with the objective of the study, as well as the known increased risks for mortality and noncommunicable diseases, a three-category dependent variable (non-overweight/obese = BMI < 24.9 kg/m^2^), overweight (BMI of 25.0–29.9 kg/m^2^) and obese (BMI ≥ 30.0 kg/m^2^) was constructed for the purposes of the current analysis.

### Independent variables

The main independent variable of interest was the duration for which women breastfed their last child in the five-year period preceding the surveys. This was categorized into five levels (not breastfed, ≤6 month, 7–12 months, 13–18 month, and >18 months), based on the number of months each woman reported to have breastfed their last child. This categorization of breastfeeding duration is consistent with commonly reported cut-offs in the literature (32, 33).

The background characteristics of women considered as relevant covariates for overweight and obesity were; age group (15–24, 25–34, 35–44 and, 45+), educational level (no education, primary, middle/junior secondary school (JSS)/junior high school (JHS) and secondary/higher education), marital status (never married, married, cohabiting and formerly married), wealth quintile (poorest, poorer, middle, rich and richest), residence (rural and urban), occupation (not working, sales/services, agriculture and manual labour), parity (1–2, 3–4 and 5+), contraceptive use (none, modern method and traditional method), and survey year (1993, 1998, 2003, 2008 and 2014).

### Statistical analysis

Both descriptive and inferential analyses were conducted using STATA 16.0 software, taking into consideration the inherent complex GDHS survey design and sample weights. The descriptive analysis involved the use of proportional distributions to describe the various background factors by duration of breastfeeding, and by overweight and obesity. Pearson's chi-squared test was used to test the statistical significance (*p* < 0.05) of the distributions. In the inferential analysis, multinomial logistic regression models were estimated to determine the association of breastfeeding duration with overweight and obesity. The choice of multinomial logistic regression was informed by the fact that the depended variable had three levels and coded as non-overweight/obese = 0, overweight = 1 and obese = 2.

Two models were estimated each for overweight and obesity, beginning with the unadjusted model with only breastfeeding duration as the candidate independent variable for inclusion (Model 1). This was followed by a multivariable model with breastfeeding duration together with the other covariates (background characteristic) included in the estimation (Model 2). This approach was employed to test the net association of breastfeeding duration with overweight and obesity, with statistical significance set at *p* < 0.05.

## Results

### Background characteristics by duration of breastfeeding

Table 1 shows results of the pooled sample of 8,516 women aged 15–49 years with complete data on breastfeeding and BMI estimates. The mean duration of breastfeeding was 14.6 (±8.2) months, while about 2% of the women reported not breastfeeding their children in the period under review. The proportion of women who breastfed increased with duration of breastfeeding, ranging from 19% among those who breastfed for up to six months to about 30% among their counterparts who breastfed for more than 18 months. The duration for which women reported to have breastfed their children varied by the background characteristics of women. A greater proportion of women who reported having breastfed for longer than 18 months were aged 54 years or more (52%), uneducated (35%), and widowed/divorced/separated (37%). They were also mostly involved in agricultural (36%) occupations and reported using hormonal contraception methods (40%). A discernible pattern of longer breastfeeding duration was observed for wealth index, parity and BMI group, with lower proportions of women reporting that they breastfed longer than 18 months as wealth, parity and BMI group increased. An inverted u-shaped pattern was observed with respect to the proportion of women breastfeeding beyond 18 months by year, peaking at 36% in the year 2003 and declining to 17% in the year 2014.

**Table 1:** Background characteristics of women by breastfeeding duration.

Characteristic	Not breastfed (*n* = 146)	≤6 mths (*n* = 1,624)	7–12 mths (*n* = 1,909)	13–18 mths (*n* = 2,372)	>18 mths (*n* = 2,465)	Total
	%	%	%	%	%	*N*
Age group						
15–24	1.3	22.7	26.0	29.1	20.8	2,164
25–34	1.5	19.7	23.0	28.1	27.6	3,963
35–44	2.2	15.4	19.0	26.8	36.6	2,091
45+	3.6	9.2	12.7	22.7	51.8	299
Educational level						
No education	1.0	19.5	21.8	22.7	35.0	3,002
Primary	2.7	18.9	21.1	27.4	29.8	1,826
Middle/JSS/JHS	1.6	18.0	22.9	33.1	24.5	3,470
Secondary/higher	4.2	31.7	34.7	19.2	10.2	218
Marital status						
Never married	2.6	22.3	25.2	30.3	19.6	390
Married	1.6	19.3	22.2	27.0	29.9	6,143
Cohabiting	1.7	21.0	24.7	29.3	23.4	1,316
Wid/div/sep	2.2	10.9	18.2	31.6	37.0	667
Wealth index						
Poorest	1.0	19.0	21.7	25.0	33.3	1,766
Poorer	1.3	17.9	22.3	24.5	34.0	1,703
Middle	1.5	19.9	21.4	28.4	28.9	1,736
Richer	2.5	19.5	22.6	29.9	25.5	1,742
Richest	2.5	19.0	24.4	31.8	22.3	1,569
Residence						
Urban	1.9	18.8	23.8	33.2	22.3	2,928
Rural	1.6	19.2	21.7	25.0	32.4	5,588
Occupation						
Not working	2.0	29.0	25.7	23.8	19.5	1,181
Prof/managerial	3.5	17.0	28.8	31.1	19.6	611
Sales/trade	1.9	16.5	22.5	32.0	27.1	2,580
Agric	1.4	18.1	20.0	24.4	36.0	3,066
Manual	1.0	18.1	21.8	30.3	28.7	1,077
Parity						
1–2	1.6	20.0	24.1	29.3	25.0	3,489
3–4	1.1	18.1	22.3	29.9	28.5	2,478
5+	2.5	18.7	20.1	23.9	34.8	2,549
Contraception						
None	1.5	22.8	23.1	25.7	26.9	6,281
Hormonal	3.1	6.9	16.7	33.3	40.0	1,062
Non-hormonal	1.8	11.7	23.5	33.8	29.2	495
Traditional	1.8	8.7	24.3	34.5	30.7	679
BMI group						
Underweight	0.6	18.0	18.5	27.9	34.9	803
Normal	1.6	18.4	22.1	27.7	30.2	5,801
Overweight	2.3	20.7	24.9	28.6	23.6	1,338
Obese	3.4	23.3	25.9	27.4	20.1	573
Survey year						
1993	1.2	21.7	24.5	29.4	23.2	1,674
1998	1.6	16.4	21.2	27.5	33.2	1,936
2003	2.1	15.7	20.2	27.5	34.5	2,143
2008	1.7	17.8	21.7	29.4	29.5	1,767
2014	2.0	29.4	27.4	23.9	17.4	995
Total	1.7	19.1	22.4	27.8	28.9	8,516

### Breastfeeding duration and background characteristics by overweight and obesity

**Figure 1:**
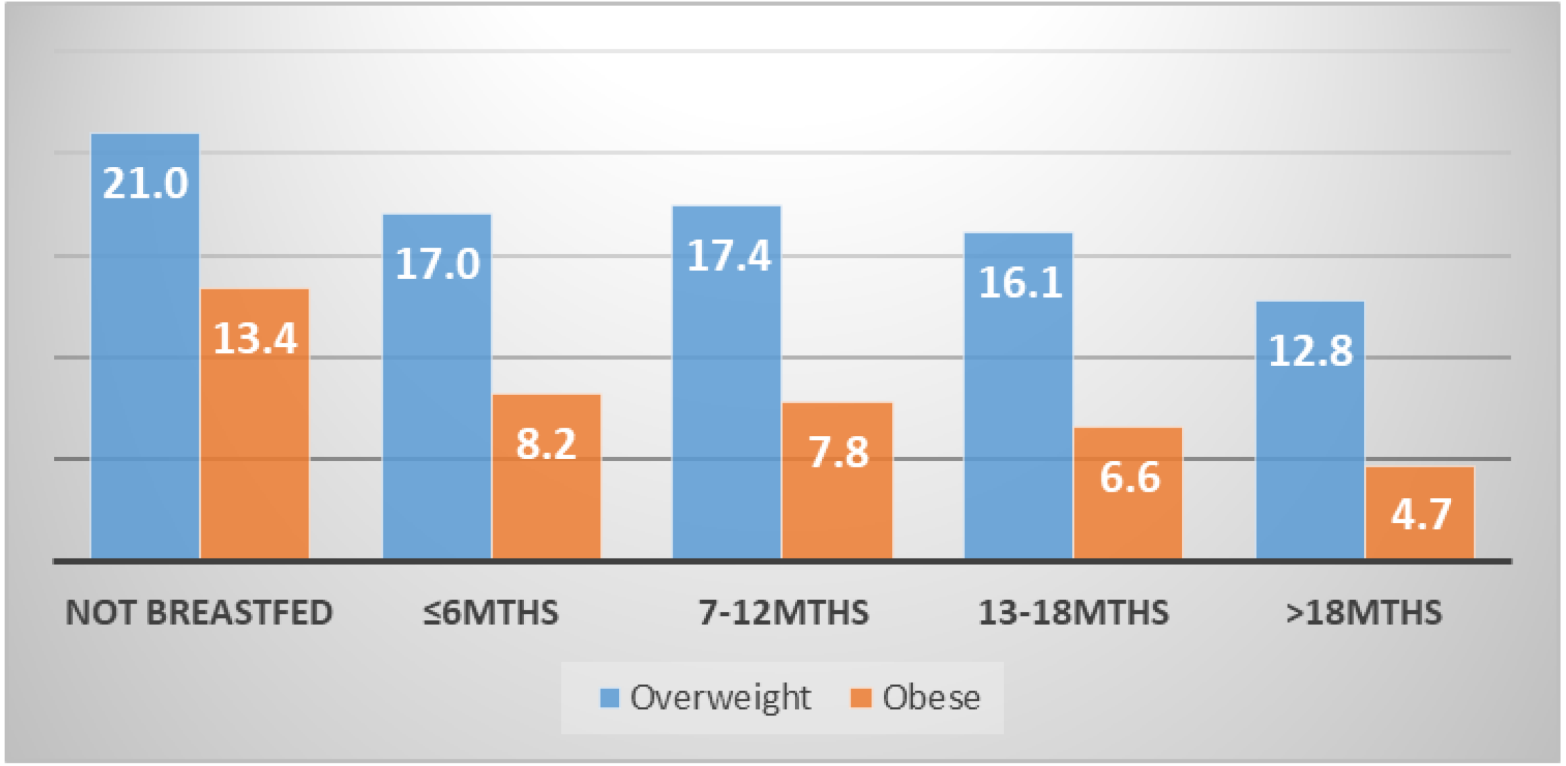
Proportional distribution of breastfeeding duration by overweight and obesity.

Table 2 shows the proportional distribution of breastfeeding duration and the background characteristics of women by overweight and obesity. Overall, more than one in five (23%) women were either overweight or obese (16% overweight vs. 7% obese). From the results, the proportion of women who were overweight and obese generally reduced with increasing duration of breastfeeding. Respectively, this reduced from about 21% and 13% among women who did not breastfeed at all to approximately 13% and 5% among those who breastfed beyond 18 months. This is further illustrated in Figure 1. The results further show wide variations in overweight and obesity across the background of women. Notably, there were generally higher proportions of both overweight and obesity among; women aged 35–44 years (18% overweight vs. 10% obese), those married (17% overweight vs. 8% obese), urban residents (25% overweight vs. 14% obese), those occupying professional/managerial roles (27% overweight vs. 14% obese), those with 3–4 children (17% overweight vs. 9% obese) and, non-hormonal contraceptive users (22% overweight vs. 12% obese). In addition, overweight and obesity increased with level of education and wealth. The results across the survey years point to an increasing trend in both overweight and obesity from 1993 (9% overweight vs. 4% obese 4%) to 2014 (24% overweight vs. 11% obese).

**Table 2:** Breastfeeding duration and background characteristics of women by overweight and obesity.

Characteristic	Overweight	Obesity	Total
	%	%	*N*
Breastfeeding duration			
Not breastfed	21.0	13.4	146
≤6 mths	17.0	8.2	1,624
7–12 mths	17.4	7.8	1,909
13–18 mths	16.1	6.6	2,372
>18 mths	12.8	4.7	2,465
Age group			
15–24	9.6	1.9	2,164
25–34	17.8	7.5	3,963
35–44	18.3	10.1	2,091
45+	13.6	7.5	299
Educational level			
No education	9.2	3.0	3,002
Primary	16.0	6.6	1,826
Middle/JSS/JHS	19.8	9.3	3,470
Secondary/higher	38.4	17.9	218
Marital status			
Never married	12.0	2.2	390
Married	16.6	7.7	6,143
Cohabiting	14.4	4.1	1,316
Wid/div/sep	12.7	5.9	667
Wealth index			
Poorest	8.3	2.3	1,766
Poorer	12.2	2.7	1,703
Middle	16.0	4.9	1,736
Richer	19.3	8.9	1,742
Richest	23.4	15.7	1,569
Residence			
Urban	24.8	14.0	2,928
Rural	11.0	2.9	5,588
Occupation			
Not working	13.7	6.3	1,181
Prof/managerial	26.5	13.9	611
Sales/trade	22.7	10.9	2,580
Agric	8.3	1.7	3,066
Manual	16.3	7.5	1,077
Parity			
1–2	15.2	5.0	3,489
3–4	16.7	9.4	2,478
5+	15.5	6.5	2,549
Contraception			
None	14.0	5.9	6,281
Hormonal	20.7	7.6	1,062
Non-hormonal	21.8	12.2	495
Traditional	19.3	9.3	679
Survey year			
1993	9.3	3.6	1,674
1998	11.2	5.0	1,936
2003	16.2	7.9	2,143
2008	21.3	8.0	1,767
2014	24.4	10.5	995
Total	15.7	6.7	8,516

### Association of breastfeeding duration with overweight and obesity

Table 3 presents results of the multinomial logistic regression analysis on the association of breastfeeding duration with overweight and obesity. The unadjusted analysis (Model 1) generally show lower odds of overweight and obesity with each consecutive longer duration of breastfeeding with reference to not breastfeeding. However, this magnitude of association was observed to be significantly more pronounced for women who breastfed for longer than a period of 18 months (overweight, OR = 0.49, 95% CI 0.398, 0.768; obesity OR = 0.28 95% CI = 0.150, 0.515). This observed association remained statistically significant only for obesity, after adjusting for other covariates in Model 2. In effect, the odds of being obese were about 0.46 (95% CI = 0.268, 0.864) and 0.41 (95% CI = 0.216, 0.764) lower for women who breastfed for 13–18 months and greater than 18 months, respectively, compared with those who did not breastfeed their children at all.

**Table 3:** Association of breastfeeding duration with overweight and obesity among women.

Characteristic	Overweight				Obesity			
	Model 1		Model 2		Model 1		Model 2	
	OR	95% CI	OR	95% CI	OR	95% CI	Characteristic	OR
Duration of breastfeeding								
Not breastfed								
≤6mths	0.712	[0.449,1.131]	1.014	[0.626,1.645]	0.539*	[0.291,0.999]	0.859	[0.457,1.614]
7–12mths	0.728	[0.460,1.154]	0.961	[0.594,1.553]	0.511*	[0.276,0.945]	0.731	[0.390,1.370]
13–18mths	0.652	[0.413,1.028]	0.750	[0.467,1.206]	0.420**	[0.228,0.775]	0.463*	[0.248,0.864]
>18mths	0.486**	[0.308,0.768]	0.672	[0.417,1.085]	0.278**	[0.150,0.515]	0.406**	[0.216,0.764]
Age group								
15–24								
25–34			1.889**	[1.510,2.363]			2.953**	[1.960,4.449]
35–44			2.319**	[1.748,3.076]			4.918**	[3.060,7.903]
45+			2.140**	[1.325,3.456]			5.965**	[2.975,11.96]
Educational level								
No education								
Primary			1.730**	[1.408,2.126]			2.166**	[1.560,3.008]
Middle/JSS/JHS			1.894**	[1.567,2.290]			2.279**	[1.696,3.062]
Secondary/higher			2.764**	[1.770,4.316]			2.478**	[1.366,4.495]
Marital status								
Never married								
Married			1.808**	[1.217,2.685]			3.005*	[1.277,7.071]
Cohabiting			1.305	[0.864,1.971]			1.530	[0.623,3.757]
Wid/div/sep			1.332	[0.836,2.124]			2.471	[0.977,6.252]
Wealth index								
Poorest								
Poorer			1.324*	[1.040,1.685]			1.020	[0.649,1.604]
Middle			1.549**	[1.211,1.980]			1.385	[0.908,2.113]
Richer			1.659**	[1.293,2.128]			1.943**	[1.274,2.965]
Richest			2.120**	[1.641,2.740]			3.385**	[2.257,5.076]
Residence								
Urban								
Rural			0.588**	[0.499,0.694]			0.374**	[0.281,0.497]
Occupation								
Not working								
Prof/managl			1.514**	[1.123,2.041]			1.438	[0.930,2.224]
Sales/trade			1.516**	[1.202,1.913]			1.348	[0.961,1.890]
Agric			0.676**	[0.524,0.872]			0.377**	[0.244,0.584]
Manual			1.113	[0.848,1.460]			1.028	[0.692,1.529]
Parity								
1–2								
3–4			1.085	[0.894,1.318]			1.700**	[1.265,2.286]
5+			1.192	[0.942,1.509]			1.449*	[1.002,2.096]
Contraception								
None								
Hormonal			1.294*	[1.053,1.589]			1.107	[0.800,1.530]
Non-hormonal			1.362*	[1.035,1.794]			1.556*	[1.072,2.258]
Traditional			1.231	[0.957,1.583]			1.272	[0.853,1.898]
Survey year								
1993								
1998			1.154	[0.912,1.461]			1.436	[0.996,2.072]
2003			1.932**	[1.551,2.407]			2.320**	[1.652,3.259]
2008			2.524**	[2.015,3.162]			2.363**	[1.660,3.365]
2014			3.075**	[2.342,4.037]			3.517**	[2.254,5.488]

Exponentiated coefficients; 95% confidence intervals in brackets; *p < .05, **p < .01.

The association of the various background characteristics of the women with overweight and obesity is largely in keeping with extant literature on the subject. As shown in Table 3, these factors similarly predict both overweight and obesity, generally showing stronger associations with obesity than overweight. With respect to age, for instance, the odds of being overweight significantly increased from about 1.9 (95% CI = 1.510, 2.363) among those aged 25–34 to about 2.1 (95% CI = 1.325, 3.456) among those aged 45 years plus; while for obesity, the odds increased from about 3.0 (95% CI = 1.960, 4.449) to 6.0 (95% CI = 2.975, 11.96) in the same respective age categories. A similar pattern was observed with respect to the association of educational level and wealth quintile with overweight and obesity, where significantly higher odds of overweight or obesity were recorded with increasing level of education and wealth. For example, the odds of obesity were over three times higher for women in the richest (OR = 3.4, 95% CI = 2.257, 5.076) wealth quintiles compared with those in the poorest. Married women also had significantly higher odds of both overweight (OR = 1.8, 95% CI = 1.217, 2.685) and obesity (OR = 3.0, 95% CI = 1.277, 7.071) compared to the never married.

By contrast, significantly lower odds of overweight (OR = 0.59, 95% CI = 0.499, 0.694) and obesity (OR = 0.37, 95% CI = 0.281, 0.497) were observed for women in rural localities compared with their urban compatriots. A similar pattern was observed for women engaged in agricultural activities, as such women had significantly lower odds of developing both overweight and obesity. Other positive associations observed show that parity (3–4 children, OR = 1.7, 95% CI = 1.265, 2.286; 5 + children, OR = 1.5, 95% CI = 1.002, 2.096) was significantly associated with only obesity; whereas contraceptive use and survey year were significantly associated with both outcomes.

## Discussion

Essentially, aggressive promotion of breastfeeding aside from influencing maternal body weights, has the potential to help achieve the Sustainable Development Goals (SDG); importantly, goals 2 (zero hunger) 3 (good health and wellbeing) and 12 (responsible consumption). Hence, this study examined the independent association of breastfeeding duration with overweight and obesity among women of reproductive age (15–49) in Ghana using multiple rounds (1993–2014) of nationally representative GDHS data. Among the pooled sample of 8,516 women who had their last birth in the five-year period preceding each of the surveys, their mean breastfeeding duration was 14.6 (±8.2). Over one-in-five (23%) of these women were either overweight or obese (16% overweight vs. 7% obese). After adjusting for other factors, the analysis revealed a significant inverse association between breastfeeding duration and obesity, with women who breastfed their last child for 13–18 months and longer than 18 months having reduced odds of being obese by 54% and 59%, respectively, compared with those who did not breastfeed their children at all.

The protective effect of longer duration of breastfeeding on obesity among women as demonstrated in this study is congruent with similar studies conducted elsewhere (20, 22, 34–36). A plausible interpretation is that breastfeeding facilitates the loss of weight gained by women through the gestational process and helps restore women's body to its pre-gestational state. Indeed, Yamamoto et al. (34) showed that full breastfeeding produced significantly more postpartum weight loss than mixed feeding or artificial feeding, while Jarlenski et al. (35) indicated that breastfeeding for at least 3 months resulted in 3.2 pounds greater weight loss for women even at 12 months postpartum.

The other covariates found to be associated with overweight and obesity largely corroborate the existing literature on the subject (37, 38). These factors similarly predict both overweight and obesity, with generally stronger effects on being obese than being overweight. Findings in this study suggest that the likelihood of being overweight or obese significantly increased with age. This pattern generally concurs with the literature (39, 40), and could be linked with the reduced metabolism associated with aging. Given that the study sample consists of women of reproductive age, this finding could partly be linked with gestational and postpartum weight gain and retention over their reproductive life course (41, 42). Indeed, this was further reflected in the results showing that women had higher probability of overweight and obesity with increasing parity.

Similarly, positive effects were seen with respect to the association of socioeconomic factors (educational level and wealth quintile) with overweight and obesity in this study, as have been widely reported in the literature (43–46). Unlike in high income countries, higher education and wealth is linked with sedentary lifestyle patterns, access to high caloric diets and reduced physical activity in low- and middle-income countries where the consumption of such diets are construed as luxuries and status symbols (47–49). Married women had significantly higher odds of both being overweight and obese than women in other categories of marital status. Although this observation is in consonance with prior studies (50–53), the plausible explanations to why married women have the tendency to be overweight or obese point towards either the selection or social causation hypotheses. The traditional social valorization of large-bodied women could result in the selection of overweight or obese women into marriage, whereas married associated improved socio-economic status and changes in social obligation, roles and expectations (childbearing and related kin support) could predispose married women to overweight and obesity.

The findings on locality of residence and occupation showing lower odds of overweight and obesity among women residing in rural areas and those engaged in agricultural occupations seem to reinforce each other. Rural folk tend to engage in agricultural activities using physically exerting and labour intensive traditional methods of production, with a sequel protective affect against overweight or obesity compared with other occupations which are typically based in urban settings (46, 54, 55). Providing further support for this is the fact that the results (Table 2) indicate a higher tendency for women involved in agriculture (36%) to breastfeed their children longer the 18 months compared with their counterparts in other occupations. The positive effect of modern contraceptive use on overweight or obesity among women was demonstrated in this study as in others (56). Nonetheless, the strong positive effect of non-hormonal contraceptive use on both overweight and obesity seemed a bit counterintuitive, especially given that they typically do not contain hormones linked with adiposity. Plausibly, women on non-hormonal contraception began with hormonal options with residual weight-gaining effects despite switching to non-hormonal types.

This study's most significant strength rests on the use of multiple rounds of internationally recognized nationally representative survey data, which was collected using robust protocols and methodological design. In addition, the large number of pooled respondents for the purposes of testing the association between breastfeeding duration provides statistical power to the estimations with minimal margin of error. Despite these strengths, the cross-sectional design based on which the data was collected presents limitations with respect to establishing temporality between breastfeeding duration and the subsequent development or otherwise of overweight and obesity. Another limitation of the data is that other important determinants of overweight and obesity such as dietary intake and physical activity patterns of the women could not be accounted for due to the absence of such information in the datasets. On the balance of these strengths and limitations, the study offers some relevant and generalizable insights on the nature of the association of breastfeeding duration with overweight and obesity among reproductive women in Ghana and perhaps beyond.

## Conclusions

The current study underscores the potential of longer breastfeeding duration in promoting optimal health and wellbeing of women by lowering their odds of developing obesity. Specifically, this study demonstrates that breastfeeding a child for a minimum of 12 months offers protection against obesity for mothers by at least 54%. Further, the study reinforces the multiple determinants of overweight and obesity among women in their reproductive age. These findings have implications for policy decision-making, including the promotion of prolonged breastfeeding among mothers as a pathway to reducing the burden of noncommunicable diseases associated with obesity among adults, while at the same time preventing childhood nutritional deficiencies and diarrheal infections. Having illuminated some of the at-risk population subgroups (age, educational, wealth status, marital status, parity etc), policy and intervention options could be tailored with such groups in mind. In particular, healthcare providers could tailor breastfeeding duration and obesity related educational messages to these subgroups of women during routine pre- and post-natal care clinic sessions.

## Data Availability

Publicly available datasets were analyzed in this study. This data can be found here: http://dhsprogram.com/data/available-datasets.cfm.

## References

[1] WHO. Infant and young child feeding. (2024). Available online at: https://www.who.int/news-room/fact-sheets/detail/infant-and-young-child-feeding#:∼:text=WHO%20and%20UNICEF%20recommend%3A,years%20of%20age%20or%20beyond (Accessed April 4, 2023).

[2] UNICEF. Nutrition: Breastfeeding. (2023). Available online at: https://data.unicef.org/topic/nutrition/breastfeeding/ (Accessed April 4, 2023).

[3] WHO. Early initiation of breastfeeding. (2024). Available online at: https://www.who.int/data/gho/indicator-metadata-registry/imr-details/early-initiation-of-breastfeeding-(-)#:∼:text=Early%20initiation%20of%20breastfeeding%2C%20within,on%20duration%20of%20exclusive%20breastfeeding (Accessed April 4, 2023).

[4] Koliaki C Dalamaga M Liatis S. Update on the obesity epidemic: after the sudden rise, is the upward trajectory beginning to flatten? Curr Obes Rep. (2023) 12:514–27. 10.1007/s13679-023-00527-y 37779155 PMC10748771

[5] WHO. Obesity and overweight. (2018). Available online at: https://www.who.int/news-room/fact-sheets/detail/obesity-and-overweight (Accessed April 15, 2023).

[6] Owobi OU Okonji OC Nzoputam CI Ekholuenetale M. Country-Level variations in overweight and obesity among reproductive-aged women in sub-saharan countries. Women. (2022) 2(4):313–25. 10.3390/women2040029

[7] GHS GSS, ICF Macro. Ghana Demographic and Health Survey 2003. Accra, Ghana: GSS, GHS, and ICF Macro (2005).

[8] GHS GSS, ICF International. Ghana Demographic and Health Survey 2014. Accra, Ghana: GSS, GHS, and ICF International (2015).

[9] ICF, GSS. Ghana Demographic and Health Survey 2022. Accra, Ghana, and Rockville. Maryland, USA: GSS and ICF (2024).

[10] WHO. One Billion People Globally Estimated to Be Living With Obesity by 2030. (2022). Available online at: https://www.worldobesity.org/resources/resource-library/world-obesity-atlas-2022 (Accessed April 16, 2023).

[11] Osunkwo DA Nguku PM Mohammed A Umeokonkwo CD Kamateeka M Ibrahim M Prevalence of obesity and associated factors in Benue State, Nigeria: a population-based study. Ann Afr Med. (2021) 20:9. 10.4103/aam.aam_36_19 33727505 PMC8102896

[12] Horta B Rollins N Dias M Garcez V Perez-Escamilla R. Systematic review and meta-analysis of breastfeeding and later overweight or obesity expands on previous study for World Health Organisation. Acta Paediatr. (2022) 112(10017):34–41. 10.1111/apa.16460 35727183

[13] Bish MR Faulks F Amir LH Huxley RR McIntyre HD James R Relationship between obesity and lower rates of breastfeeding initiation in regional Victoria, Australia: an 8-year retrospective panel study. BMJ Open. (2021) 11(2):e044884. 10.1136/bmjopen-2020-044884 33568376 PMC7878145

[14] Rooney BL Schauberger CW. Excess pregnancy weight gain and long-term obesity: one decade later. Obstet Gynecol. (2002) 100(2):245–52. 10.1016/s0029-7844(02)02125-7 12151145

[15] Mannan M Doi SA Mamun AA. Association between weight gain during pregnancy and postpartum weight retention and obesity: a bias-adjusted meta-analysis. Nutr Rev. (2013) 71(6):343–52. 10.1111/nure.12034 23731445

[16] Stuebe AM Rich-Edwards JW. The reset hypothesis: lactation and maternal metabolism. Am J Perinatol. (2009) 26(01):081–8. 10.1055/s-0028-1103034 19031350 PMC3006166

[17] Rasmussen KM Yaktine AL. Weight Gain During Pregnancy: Reexamining the Guidelines. Washington, DC, USA: National Academies Press (US) (2009). 20669500

[18] Butte NF King JC. Energy requirements during pregnancy and lactation. Public Health Nutr. (2005) 8(7a):1010–27. 10.1079/PHN2005793 16277817

[19] Keyes M Andrews C Midya V Carrasco P Guxens M Jimeno-Romero A Mediators of the association between maternal body mass index and breastfeeding duration in 3 international cohorts. Am J Clin Nutr. (2023) 118(1):255–63. 10.1016/j.ajcnut.2023.04.004 37407164 PMC10493413

[20] Cieśla E Stochmal E Głuszek S Suliga E. Breastfeeding history and the risk of overweight and obesity in middle-aged women. BMC Women’s Health. (2021) 21(1):1–9. 10.1186/s12905-020-01152-w 33975572 PMC8114504

[21] Bobrow KL Quigley MA Green J Reeves GK Beral V. Persistent effects of women’s parity and breastfeeding patterns on their body mass index: results from the million women study. Int J Obes. (2013) 37(5):712–7. 10.1038/ijo.2012.76 22777544 PMC3647235

[22] Tahir MJ Haapala JL Foster LP Duncan KM Teague AM Kharbanda EO Association of full breastfeeding duration with postpartum weight retention in a cohort of predominantly breastfeeding women. Nutrients. (2019) 11(4):938. 10.3390/nu11040938 31027268 PMC6520964

[23] Perez MR de Castro LS Chang Y-S Sañudo A Marcacine KO Amir LH Breastfeeding practices and problems among obese women compared with nonobese women in a Brazilian hospital. Women’s Health Report. (2021) 2(1):219–26. 10.1089/whr.2021.0021 PMC8243705 34235509

[24] Bever Babendure J Reifsnider E Mendias E Moramarco MW Davila YR. Reduced breastfeeding rates among obese mothers: a review of contributing factors, clinical considerations and future directions. Int Breastfeed J. (2015) 10(21):1–11. 10.1186/s13006-015-0046-5 26140049 PMC4488037

[25] Mullaney L O’Higgins AC Cawley S Kennedy R McCartney D Turner MJ. Breast-feeding and postpartum maternal weight trajectories. Public Health Nutr. (2016) 19(8):1397–404. 10.1017/S1368980015002967 26466770 PMC10271137

[26] Neville CE McKinley MC Holmes VA Spence D Woodside JV. The relationship between breastfeeding and postpartum weight change–a systematic review and critical evaluation. International Journal Obesity. (2014) 38(4):577–90. 10.1038/ijo.2013.132 23892523

[27] Oken E Patel R Guthrie LB Vilchuck K Bogdanovich N Sergeichick N Effects of an intervention to promote breastfeeding on maternal adiposity and blood pressure at 11.5 y postpartum: results from the promotion of breastfeeding intervention trial, a cluster-randomized controlled trial. Am J Clin Nutr. (2013) 98(4):1048–56. 10.3945/ajcn.113.065300 23945719 PMC3778859

[28] Hauff LE Leonard SA Rasmussen KM. Associations of maternal obesity and psychosocial factors with breastfeeding intention, initiation, and duration. Am J Clin Nutr. (2014) 99:524–34. 10.3945/ajcn.113.071191 24401717 PMC3927688

[29] Measure DHS. Available datasets: Ghana. (2023). Available online at: https://dhsprogram.com/data/available-datasets.cfm (Accessed March 15, 2023).

[30] ICF International. The Demographic and Health Surveys (DHS) Program. (2012). Available online at: https://www.icf.com/clients/health/demographic-health-surveys-technical-assistance (Accessed March 15, 2023).

[31] WHO. Body mass index-BMI. (2000). Available online at: https://www.who.int/europe/news-room/fact-sheets/item/a-healthy-lifestyle—who-recommendations (Accessed March 16, 2023).

[32] Lopez DA Foxe JJ Mao Y Thompson WK Freedman EG. Breastfeeding duration is associated with domain-specific improvements in cognitive performance in 9–10-year-old children. Front Public Health. (2021) 9(1):657422. 10.3389/fpubh.2021.657422 33981668 PMC8109433

[33] Walfisch A Sermer C Cressman A Koren G. Breast milk and cognitive development—the role of confounders: a systematic review. BMJ Open. (2013) 3(8):e003259. 10.1136/bmjopen-2013-003259 23975102 PMC3753522

[34] Yamamoto M Takami M Misumi T Kawakami C Miyagi E Ito S Effects of breastfeeding on postpartum weight change in Japanese women: the Japan environment and children’s study (JECS). Plos One. (2022) 17(5):e0268046. 10.1371/journal.pone.0268046 35507607 PMC9067657

[35] Jarlenski MP Bennett WL Bleich SN Barry CL Stuart EA. Effects of breastfeeding on postpartum weight loss among US women. Prev Med. (2014) 69:146–50. 10.1016/j.ypmed.2014.09.018 25284261 PMC4312189

[36] Mantzorou M Papandreou D Vasios GK Pavlidou E Antasouras G Psara E Exclusive breastfeeding for at least four months is associated with a lower prevalence of overweight and obesity in mothers and their children after 2–5 years from delivery. Nutrients. (2022) 14(17):3599. 10.3390/nu14173599 36079855 PMC9459704

[37] Jiang M Gao H Vinyes-Pares G Yu K Ma D Qin X Association between breastfeeding duration and postpartum weight retention of lactating mothers: a meta-analysis of cohort studies. Clin Nutr. (2018) 37(4):1224–31. 10.1016/j.clnu.2017.05.014 28606701

[38] Alves MDS Almeida MAM Gomes CDB Ferrari AP Parada CMGDL Carvalhaes MADBL. Longer duration of exclusive breastfeeding reduces maternal weight retention: results from the CLaB study. Revista Brasileira de Saúde Materno Infantil. (2020) 20:273–84. 10.1590/1806-93042020000100015

[39] Adesina AF Peterside O Anochie I Akani NA. Weight status of adolescents in secondary schools in port harcourt using body mass Index (BMI). Ital J Pediatr. (2012) 38(1):31. 10.1186/1824-7288-38-31 22823927 PMC3412731

[40] Poobalan AS Aucott LS Clarke A Smith WCS. Physical activity attitudes, intentions and behaviour among 18 to 25 year olds: a mixed method study. BMC Public Health. (2012) 12(1):640. 10.1186/1471-2458-12-640 22892291 PMC3490897

[41] Dalrymple KV Uwhubetine O Flynn AC Pasupathy D Briley AL Relph SA Modifiable determinants of postpartum weight loss in women with obesity: a secondary analysis of the UPBEAT trial. Nutrients. (2021) 13(6):1979. 10.3390/nu13061979 34207523 PMC8227672

[42] Hollis JL Crozier SR Inskip HM Cooper C Godfrey KM Harvey NC Modifiable risk factors of maternal postpartum weight retention: an analysis of their combined impact and potential opportunities for prevention. Int J Obes. (2017) 41(7):1091–8. 10.1038/ijo.2017.78 PMC5500180 28337028

[43] Dinsa GD Goryakin Y Fumagalli E Suhrcke M. Obesity and socioeconomic status in developing countries: a systematic review. Obes Rev. (2012) 13(11):1067–79. 10.1111/j.1467-789X.2012.01017.x 22764734 PMC3798095

[44] Doku DT Neupane S. Double burden of malnutrition: increasing overweight and obesity and stall underweight trends among Ghanaian women. BMC Public Health. (2015) 15(1):670. 10.1186/s12889-015-2033-6 26178521 PMC4502461

[45] Neuman M Kawachi I Gortmaker S Subramanian SV. Urban-rural differences in BMI in low- and middle-income countries: the role of socioeconomic status. Am J Clin Nutr. (2013) 97(2):428–36. 10.3945/ajcn.112.045997 23283503 PMC3742298

[46] Tuoyire DA Kumi-Kyereme A Doku DT. Socio-demographic trends in overweight and obesity among parous and nulliparous women in Ghana. BMC Obes. (2016) 3(1):44. 10.1186/s40608-016-0124-2 27826451 PMC5093993

[47] Victora CG Bahl R Barros AJ França GV Horton S Krasevec J Breastfeeding in the 21st century: epidemiology, mechanisms, and lifelong effect. Lancet. (2016) 387(10017):475–90. 10.1016/S0140-6736(15)01024-7 26869575

[48] Cohen E Boetsch G Palstra FP Pasquet P. Social valorisation of stoutness as a determinant of obesity in the context of nutritional transition in Cameroon: the bamiléké case. Soc Sci Med. (2013) 96:24–32. 10.1016/j.socscimed.2013.07.004 24034948

[49] Renzaho AM. Fat, rich and beautiful: changing socio-cultural paradigms associated with obesity risk, nutritional status and refugee children from sub-saharan Africa. Health Place. (2004) 10(1):105–13. 10.1016/S1353-8292(03)00051-0 14637290

[50] Dogan N Toprak D Demir S. Prevalence of obesity and associated risk factors in afyonkarahisar-Turkey. Türkiye clinics. Journal of Medical Sciences. (2011) 31(1):122. 10.5336/medsci.2009-14564

[51] Brenner DR Poirier AE Haig TR Akawung A Friedenreich CM Robson PJ. Measures of excess body weight and anthropometry among adult albertans: cross-sectional results from Alberta’s tomorrow project cohort. BMC Public Health. (2017) 17(1):1–11. 10.1186/s12889-017-4887-2 29178858 PMC5702087

[52] Sarma H Saquib N Hasan MM Saquib J Rahman AS Khan JR Determinants of overweight or obesity among ever-married adult women in Bangladesh. BMC Obes. (2016) 3:1–11. 10.1186/s40608-016-0093-5 26962459 PMC4774107

[53] Şahin T Borlu A. Prevalence of obesity in women of reproductive age group and related factors. A study from southeastern Turkey. Niger J Clin Pract. (2022) 25(6):801–8. 10.4103/njcp.njcp_1587_21 35708421

[54] Bell AC Adair LS Popkin BM. Understanding the role of mediating risk factors and proxy effects in the association between socio-economic status and untreated hypertension. Soc Sci Med. (2004) 59(2):275–83. 10.1016/j.socscimed.2003.10.028 15110419

[55] Abdulai A. Socio-economic characteristics and obesity in underdeveloped economies: does income really matter? Appl Econ. (2010) 42(2):157–69. 10.1080/00036840701604313

[56] Grimes DA Lopez LM O’Brien P Raymond EG (2009) Progestin-only pills for contraception. In Grimes DA editor, Cochrane database of systematic reviews. Chichester, UK: John Wiley & Sons, Ltd. 10.1002/14651858.CD007541

